# Probability Distributions with Singularities

**DOI:** 10.3390/e21030312

**Published:** 2019-03-21

**Authors:** Federico Corberi, Alessandro Sarracino

**Affiliations:** 1 Dipartimento di Fisica “E. R. Caianiello”, Università di Salerno, via Giovanni Paolo II 132, 84084 Fisciano (SA), Italy; 2 INFN, Gruppo Collegato di Salerno, and CNISM, Unità di Salerno, Università di Salerno, via Giovanni Paolo II 132, 84084 Fisciano (SA), Italy; 3 Dipartimento di Ingegneria, Università della Campania “L. Vanvitelli”, via Roma 29, 81031 Aversa (CE), Italy

**Keywords:** large deviations, phase transitions, condensation of fluctuations, fluctuation relations

## Abstract

In this paper we review some general properties of probability distributions which exhibit a singular behavior. After introducing the matter with several examples based on various models of statistical mechanics, we discuss, with the help of such paradigms, the underlying mathematical mechanism producing the singularity and other topics such as the condensation of fluctuations, the relationships with ordinary phase-transitions, the giant response associated to anomalous fluctuations, and the interplay with fluctuation relations.

## 1. Introduction

Quantitative predictions on the occurrence of rare events can be very useful particularly when these events can produce macroscopic effects on the system. This occurs, for instance, when a large fluctuation triggers the decay of a metastable state [1] leading the system to a completely different thermodynamic condition. Other examples with rare deviations producing important effects are found in many other contexts, as in information theory [2] and finance [3].

For a collective variable *N*, namely a quantity formed by the addition of many microscopic contributions, such as the energy of a perfect gas or the mass of an aggregate, typical fluctuations are regulated by the central limit theorem. Rare events, instead, may go beyond the theorem’s validity and are described by large deviations theory [4,5] which, in principle, aims at describing the whole spectrum of possible fluctuations, no matter how large or rare they are, by means of their full probability distribution P(N).

It has been found that, in many cases, P(N) exhibits a singular behavior, in that it is non-differentiable around some value (or values) $N_{c}$ of the fluctuating variable [3,6,7,8,9,10,11,12,13,14,15,16,17,18,19,20,21,22,23,24,25,26,27,28,29,30,31,32,33,34,35,36,37,38,39]. Such singularities have an origin akin to those observed in the thermodynamic potentials of systems at criticality. Indeed, a correspondence can be shown between P(N) and the free energy of a companion system, related to the one under study by a duality map [4,34,35,36], which is interested by a phase-transition.

Recently, a great effort has been devoted to the characterization of these singular behaviors in the large deviation functions of different models where analytical results can be obtained. This has unveiled a rich phenomenology which shares common features. In most cases non-analyticities are a consequence of a particular condensation phenomenon denoted as condensation of fluctuations.

It occurs when a significant contribution to the fluctuations is built within a limited part of phase-space, or is provided by just one of the degrees of freedom of the system. This is analogous to what happens, for instance, in the usual condensation of a gas when it concentrates in a liquid drop, or in the well-known Bose–Einstein condensation, where the mode with vanishing wavevector contributes macroscopically. However, while usual condensation represents the typical behavior of the system, the condensation of fluctuations can only be observed when certain rare events take place.

Another interesting feature of systems with singular probability distributions can be their extreme sensibility to small perturbations. Usually, the properties of a system made of many constituents or degrees of freedom do not change much if some features of a single particle are slightly changed. This is true both for the average properties and for the fluctuations. For instance, neither the average energy of a gas nor its fluctuations change appreciably if the mass of one single molecule is increased a bit. This is simply because this particle is only one out of an Avogadro number. However, when condensation of fluctuations occurs, one can observe a giant response if the perturbed degree of freedom is exactly the one that contributes macroscopically to the fluctuation.

Singular probability distributions raise the question about the validity of the fluctuation relations (FRs). These relations have been extensively studied recently [40,41] because they reflect general symmetries of the deviations of certain quantities and are believed to contribute to a general understanding of non-equilibrium states. In particular, FRs connect the probability of observing events with a certain value *N* of the fluctuating variable, to the probability of the events associated to the opposite value −N. Among other open issues on the subject, one is represented by the case of singular fluctuations. Indeed, the singularity in $N_{c}$ usually separates two regions where fluctuations have very different properties. For instance, on one side of $N_{c}$ one can have a standard situation where all the degrees of freedom contribute, whereas on the other side fluctuations can condense and be determined by the contribution of a single degree. Clearly, if *N* is such that *N* and −N fall on different branches of P(N), namely on the two sides of $N_{c}$, the mechanism whereby an FR can be fulfilled must be highly non-trivial. In general, singular probability distributions may, or may not, exhibit the FR and a general understanding of this point is still not achieved.

This paper is a brief review devoted to the discussion of singular probability distributions where, without any presumption of neither completeness or mathematical rigor, we present examples of models where such non-analyticities show up, we highlight the mathematical mechanism producing condensation, and we discuss some relevant aspects related to the subject, such as those mentioned above. We do that in a physically oriented spirit, providing whenever possible an intuitive interpretation and a simple perspective. Non-differentiable probability distributions have been previously reviewed also in [42], where however the authors focus on different models and complementary aspects with respect to those addressed in this paper.

The paper is organized as follows. In Section 2 we recall some basic results of probability theory and introduce some notations. In Section 3 we present some models of statistical mechanics where non-differentiable probability distributions have been computed for different collective quantities. In Section 4 we illustrate in detail some phenomena related to the singular distribution function, mainly using the urn model as a paradigm, and discuss how similar behaviors arise in other systems. We also discuss the topic of the fluctuation relations. More specific features, such as giant response and observability, are then presented in Section 5, and, finally, some conclusions are drawn in Section 6.

## 2. Probability Distributions: Generalities

We consider a generic stochastic system, whose physical state is defined by the random variable *x* taking values on a suitable phase space. We will be mainly interested in the behavior of collective random variables, that are defined as the sum of a large number of microscopic random variables. For these quantities some general results can be derived [5]. As an example let us consider the sum $N=\sum_{j=1}^{M}x_{j}$ of a sequence of *M* random variables $x_{j}$, with empirical mean

(1) $$\rho =\frac{N}{M}=\frac{1}{M}\sum_{j=1}^{M}x_{j}.$$

The quantities $x_{j}$ can represent a sequence of states of a system (for instance, the position of a particle along a trajectory) or an ensemble of variables describing its microscopic constituents (e.g., the energies of the single particles of a gas). In the case of independent identically distributed variables, with expectation 〈x〉 and finite variance σ, one has that the empirical mean tends to 〈x〉 for large *M*, namely

(2) $$\lim_{M\to \infty }p\left(\rho -\langle x\rangle <\epsilon \right)\to 1,$$

where ϵ is a small quantity and hereafter p(E) (also P(E) or P(E)) is the probability of an event *E*. The above equation represents the Law of Large Numbers.

As a further step, one can describe the statistical behavior of the small fluctuations of ρ around the average 〈ρ〉, δρ=ρ−〈ρ〉, introducing the quantity

(3) $$z_{M}=\frac{1}{\sigma \sqrt{M}}\sum_{j=1}^{M}\left(x_{j}-\langle x\rangle \right),$$

which, for very large *M*, and for $\delta \rho ≲O(\sigma /\sqrt{M})$, has the following distribution function

(4) $$p\left(z_{M}=z\right)≃\frac{1}{\sqrt{2\pi }}e^{-\frac{z^{2}}{2}}.$$

This result is the central limit theorem (CLT), that holds also in the case of weakly correlated variables.

More in general, fluctuations of arbitrary size of the quantity ρ can, under certain conditions, be characterized by the large deviation principle (LDP)

(5) $$p\left(\rho =y\right)∼e^{-MI(y)},$$

where I(y) is the so called rate function. When p(ρ) has a single absolute maximum (in 〈ρ〉), the rate function is positive everywhere but for y=〈ρ〉, where it vanishes. It is easy to obtain the CLT Equation (4) from the LDP Equation (5) by expanding up to second order the function I(y) around 〈ρ〉. However, as we will discuss in detail below, there are interesting cases where the LDP in the form Equation (5) is not satisfied.

A simple example where LDP holds and the rate function can be easily computed is obtained by considering $\left\{x_{j}\right\}$ as dichotomous variables taking the value +1 with probability *q* and −1 with probability 1−q. Then, using the Stirling approximation, one obtains the explicit expression for the rate function:

(6) $$I\left(y\right)=\frac{1+y}{2}\ln\frac{1+y}{2q}+\frac{1-y}{2}\ln\frac{1-y}{2(1-q)}.$$

Expanding Equation (6) around the mean 〈y〉=2q−1 one has the CLT

(7) $$I\left(y\right)≃\frac{{\left(y-\langle y\rangle \right)}^{2}}{2(1-\langle y\rangle )}.$$

## 3. Singular Probability Distributions: Examples

As far as small deviations of a collective variable are considered, the associated probability distribution is usually regular, being a Gaussian when the hypotheses of the CLT are satisfied. Moving to the realm of large deviations, instead, can hold surprises as, for instance, the emergence of non-analyticities. Before deepening the meaning and the bearings of the singular behavior, in this section we first itemize some examples of systems where it has been observed. We will then study it in more detail in some specific models in the following sections.

### 3.1. Gaussian Model

The Gaussian model is a reference model of statistical mechanics. An order-parameter field $\phi (\vec{x})$ (which in the magnetic language can be thought of as a local magnetization at site $\vec{x}$) is ruled by the following Hamiltonian

(8) $$H\left[\varphi \right]=\frac{1}{2}\int_{V}d\vec{x}\;\left[{\left(\nabla \varphi \right)}^{2}+r\varphi^{2}\left(\vec{x}\right)\right],$$

where r>0 is a parameter and *V* the volume. This simple model can be exactly solved and has a rather trivial phase-diagram without phase transitions.

**Figure 1:**
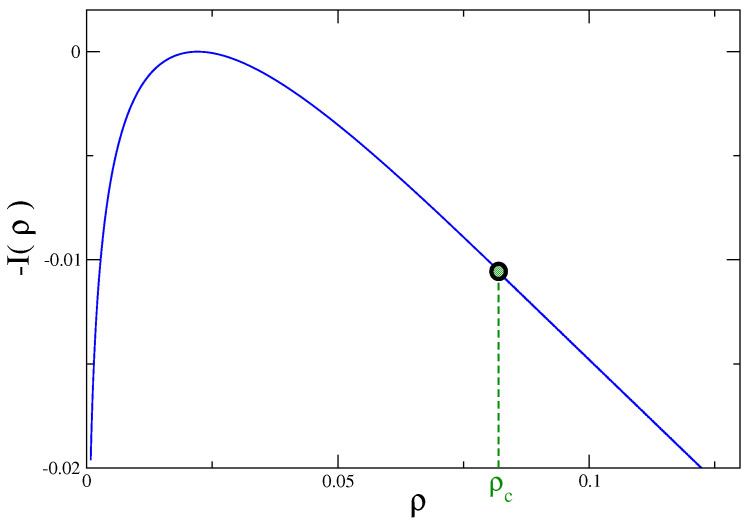
The (negative) rate function I(ρ) of the variance *N* of the order parameter field in the Gaussian model in d=3, with r=1, in equilibrium at the temperature T=0.2.

Let us consider the collective variable

(9) $$N\left[\varphi \right]=\int_{V}d\vec{x}\;\varphi^{2}\left(\vec{x}\right),$$

namely the order parameter variance, and its density ρ=N/V. Its probability distribution was computed analytically in [34,35,36]. The (negative) rate function of this quantity, evaluated in equilibrium at a given temperature *T*, is plotted in Figure 1. The curve has a maximum in correspondence to the most probable value, where I(ρ) vanishes. Far from such maximum, in the large deviations regime, the rate function exhibits a singularity (marked with a green dot) at $\rho =\rho_{c}$. In this point the third derivative of the rate function has a discontinuity [34,35,36]. The existence of such a singularity is related to the fact that, as we will discuss later, fluctuations with $\rho >\rho_{c}$ have a different character with the respect to the ones in the region $\rho <\rho_{c}$ where the average, or typical, behavior of the system (i.e., the most probable value of ρ) is located.

### 3.2. Large-N Model

Another reference model of statistical mechanics is the description of a magnetic system in terms of the Ginzburg–Landau Hamiltonian

(10) $$H\left[\varphi \right]=\frac{1}{2}\int_{V}d\vec{x}\;\left[{\left(\nabla \varphi \right)}^{2}+r\varphi^{2}\left(\vec{x}\right)+\frac{g}{2N}{\left(\varphi^{2}\right)}^{2}\right],$$

where the N-components vectorial field φ has a meaning similar to that of the Gaussian model, and r<0 and g>0 are parameters. In the large-N limit (sometimes also denoted as the spherical limit) the model is exactly soluble. There is a phase transition at a finite critical temperature $T_{c}$ separating a paramagnetic phase for $T>T_{c}$ from a ferromagnetic one at $T<T_{c}$.

**Figure 2:**
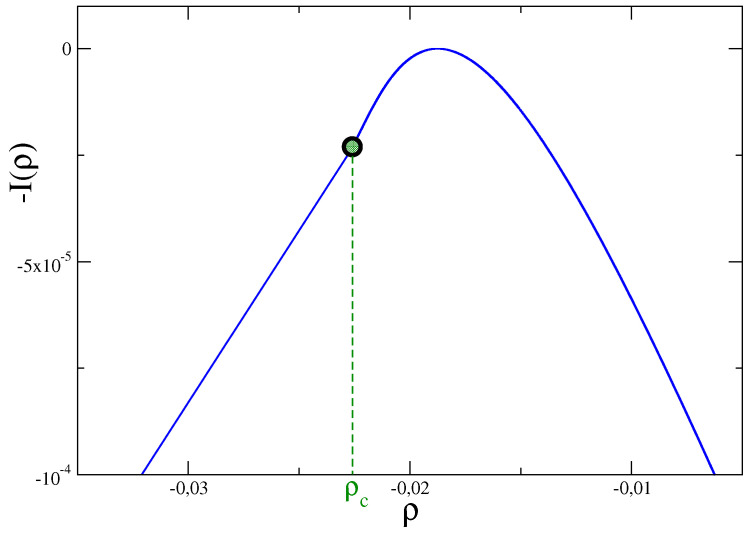
The (negative) rate function I(ρ) of the probability distribution P(N) of the energy *N* exchanged by the large-N model in d=3, with g=−r=1, with the environment after a quench to zero temperature.

The probability distribution of the energy $N\left(t,t_{w}\right)=H\left[\varphi ,t\right]-H\left[\varphi ,t_{w}\right]$ exchanged by the system in a time interval $\left[t_{w},t\right]$ with a thermal bath was computed exactly in [37]. The (negative) rate function of the intensive quantity $\rho \left(t,t_{w}\right)=N\left(t,t_{w}\right)/V$ is shown in Figure 2. This figure refers to the case of a system quenched from a very high temperature to another $T<T_{c}$. Also in this case there is a singularity corresponding to a certain value the quantity $\rho \left(t,t_{w}\right)=\rho_{c}$ where the third derivative has a discontinuity, and this reflects a different mechanism of heat exchanges for $\rho <\rho_{c}$ and for $\rho >\rho_{c}$.

### 3.3. Urn Model

**Figure 3:**
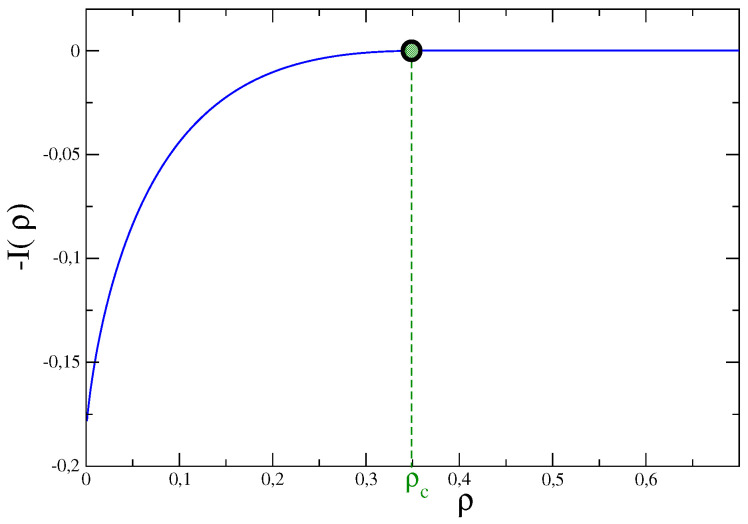
The rate function I(ρ) of the probability distribution P(N) of the total number of particles *N* in the urn model with k=3.

Let us consider a set of integer variables $n_{i}\geq 0$ (i=1,⋯,M) equally distributed in such a way that the probability of having a certain value *n* of $n_{i}$ is

(11) $$p\left(n\right)=\zeta^{-1}{\left(n+1\right)}^{-k},$$

where ζ is a normalization constant and *k* a parameter. One can think of having *M* urns, each of them hosts a quantity $n_{m}$ of particles taken with probability Equation (11) from a reservoir. This setting is appropriate to describe a wealth of situations in many areas of science, from network dynamics to financial data. The probability distribution of the total number of particles

(12) $$N=\sum_{m=1}^{M}n_{m}$$

was studied for large *M* in different contexts [14,17,21,22,23,43]. The (negative) rate function is shown in Figure 3. Also in this model it is found that, if k>2, there is a singularity at $\rho =\rho_{c}$, that in this particular case coincides with the average value 〈ρ〉. Notice that in this case, at variance with the previous examples, the rate function vanishes in the whole region $\rho \geq \rho_{c}$. This is due to the fact that P(ρ) has a weaker dependence on *M* with respect to the exponential one of Equation (5), and hence the LDP is violated for $\rho >\rho_{c}$. We will comment later on that.

### 3.4. Stochastic Maxwell-Lorentz Particle Model

The so-called stochastic Maxwell–Lorentz gas [44,45] consists of a probe particle of mass *m* whose velocity *v* changes due to the collisions with bath particles, of mass *M* at temperature *T*, and due to the acceleration produced by an external force field E. Collisions with the scatterers change instantaneously the probe’s velocity from *v* to $v^{\prime }$ and we assume the simple collision rule $v^{\prime }=V$, where *V* is the velocity of the scatterer, drawn from a Gaussian distribution:

(13) $$P_{\mathrm{scatt}}\left(V\right)=\sqrt{\frac{M}{2\pi T}}e^{-\frac{MV^{2}}{2T}}.$$

The scatterers play the role of a thermal bath in contact with the probe particle. This model is a particular case of a more general class of systems studied in [44,45]. During a time τ between two consecutive collisions, the probe performs a deterministic motion under the action of the field E. We assume that the duration of flight times τ is exponentially distributed $P_{\tau }\left(\tau \right)=\frac{1}{\tau_{c}}\exp\left(-\tau /\tau_{c}\right)$ and independent of the relative velocity of the particles. The system reaches a non-equilibrium stationary state characterized by a total entropy production $\Delta s_{\mathrm{tot}}$, associated with the velocity v(t), defined as

(14) $$\Delta s_{\mathrm{tot}}\left(t\right)=\ln\frac{P({\left\{v\left(s\right)\right\}}_{0}^{t})}{P(\bar{{\left\{v\left(s\right)\right\}}_{0}^{t}})},$$

where $P({\left\{v\left(s\right)\right\}}_{0}^{t})$ and $P(\bar{{\left\{v\left(s\right)\right\}}_{0}^{t}})$ are, respectively, the pdf of a path ${\left\{v\left(s\right)\right\}}_{0}^{t}$ spanning the time interval [0,t] and of the time-reversed path $\bar{{\left\{v\left(s\right)\right\}}_{0}^{t}}={\left\{-v\left(t-s\right)\right\}}_{0}^{t}$ [46]. This fluctuating quantity takes contributions at any time and is therefore extensive in *t*. In this example it plays the role of the collective variable *N*, and *t* plays the role of the number *M* of elements contributing to it.

**Figure 4:**
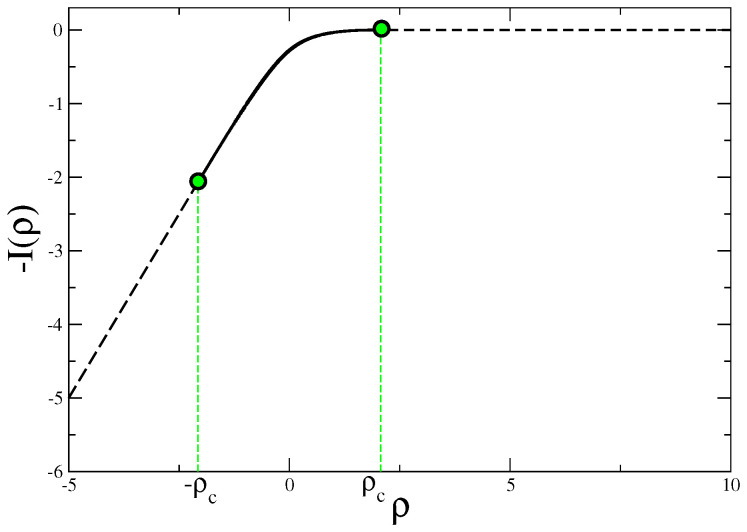
The rate function I(ρ) of the quantity $\rho =\Delta s_{\mathrm{tot}}/t$ for the Maxwell–Lorentz gas model [11], computed analytically in the limit t→∞.

The rate function I(ρ) of the quantity $\rho =\Delta s_{\mathrm{tot}}/t$ was studied in [11] by means of numerical simulations for finite times and analytically in the limit t→∞. This quantity is shown in Figure 4, where $\rho_{c}=m\tau_{c}E^{2}/\theta$, with θ=Tm/M playing the role of an effective temperature [47]. Also in this case, as for the urn model, I(ρ) vanishes and $P(\Delta s_{tot})$ does not satisfy a standard LDP for $\rho >\rho_{c}$. Indeed it can be shown that the far positive tail of $P(\Delta s_{tot})$ scales exponentially with $\sqrt{t}$ rather than with *t* [11], how it should be if the LDP (Equation (5)) holds. Recently, the nature of the singularities in I(ρ) and their physical meaning have been thoroughly discussed in a similar model in [48], where the observed non-analytical behaviors have been related to a first-order dynamical phase transition.

### 3.5. Some Other Models

We have discussed above some models where a singular probability distribution was found. All these cases can be grouped into two classes: the first contains the cases where the rate function is well defined, although it contains some non-analyticity point. The examples of Section 3.1 and Section 3.2 behave in this way. The second class is the one represented by the urn model, where the probability distribution is still singular, but the the rate function is not defined in a certain region (that is to say it vanishes identically). The Maxwell–Lorentz gas is an example where the two behaviors are exhibited in different regions of the fluctuation spectrum.

Beyond the cases discussed before, other examples of singular behavior include the probability distribution of the work done by active particles [38], of the heat exchanged by harmonic oscillators during a quench with a thermal bath [39], of the magnetization in the spherical model [6,7], of the displacement of a Brownian walker with memory [10], of the work done in a quantum quench [12], and many others [4,25,26,27,28,29,30,31,32,33].

We also mention the case where the singularity appears as a “kink” at zero in the probability distribution, showing a linear regime for negative values. This behavior has been observed in the distribution of the entropy production and of other currents for a driven particle in periodic potentials [49,50,51], in a molecular motor model, described in [52], and in the experimental results reported in [53], where the large deviation function of the velocity of a granular rod was measured. In general, the presence of the kink can be related to different physical mechanisms [54], such as intermittency [55], detailed fluctuation theorem [56], and dynamical phase transitions [57].

## 4. General Features of Singular Probability Distributions

In this section we will discuss some general properties of singular probability distributions observed in the different models mentioned above, focusing on the common physical interpretation and on the underlying mathematical structure.

### 4.1. Duality

The singular behavior of the probability distribution seen in the examples of the previous section has an interpretation akin to the occurrence of phase transitions in ordinary critical phenomena. In order to discuss this point we can refer to the Gaussian model as a paradigm. The partition function is

(15) Z=∫δφP(φ),

where P is the probability of microscopic configurations as specified by the field φ. For instance, in a canonical setting it is $P\left(\varphi \right)=Z^{-1}\exp\left[-\beta H\left(\varphi \right)\right]$, where β is the inverse temperature $\beta =1/(k_{B}T)$; in this case *Z* depends on *T* and *V*, the volume. On the other hand the probability of the collective variable *N* of Equation (9) can be written as

(16) $$P\left(N\right)=\int \delta \varphi \;P\left(\varphi \right)\;\delta \left(\int_{V}d\vec{x}\varphi^{2}\left(\vec{x}\right)-N\right).$$

In view of Equation (15), one can recognize Equation (16) as a partition function as well. However this is not the partition function of the original model that is, in this example, the Gaussian one. Instead, P(N) in Equation (16) can be interpreted as the partition function of a dual system that can be obtained from the original one upon removing all the configurations such that the argument of the delta function in Equation (16) does not vanish. In other words, this is the model one arrives at upon constraining configurations in a certain way. In this case the requirement is that the variance of φ must equal a given value *N*. Such a system, a Gaussian model with a constraint on the variance, is the spherical model of Berlin and Kac [58].

The equilibrium properties of the spherical model are exactly known. For fixed *N*, there is a phase-transition at a critical temperature $T_{c}$, from a disordered phase for $T\geq T_{c}$ to an ordered one below $T_{c}$. Equivalently, still in the Berlin–Kac model, if one keeps *T* fixed, the transition occurs changing the variance N[φ] defined in Equation (9) upon crossing a critical value $N_{c}$. The ordered phase is found for $N>N_{c}$, in this case. The presence of such a phase transition crossing $N_{c}$ determines a singularity of the partition function P(N) of the spherical model (Equation (16)) at $N=N_{c}$. However the same quantity P(N) is also the probability distribution of the quantity N[φ] in the context originally considered, the Gaussian model. This explains what one observes in Figure 1. $N_{c}$ is the value of *N* marked by a dot in this figure, where the singular behavior shows up.

This dual interpretation of P(N), as a probability distribution of a collective variable in the original model, or as a partition function in a dual model, may help to understand why singularities are manifested in the probability distributions. Indeed, if one asks the question: why a simple model without phase transitions, such as the Gaussian model, exhibits a non trivial singularity in the probability distribution P(N), the answer can be that, although the original model is quite simple, the dual one is far from being trivial, with a phase-transition induced by the presence of the constraint. This generates anomalous behavior in the fluctuation spectrum of the original model.

We have discussed the fact that imposing a constraint to the Gaussian model we change the system into a dual one that is interested by a phase transition, since this is the spherical model. Is this an isolated example or has this feature some generality? The answer is that it occurs quite often. Besides the above mentioned spherical model, another well known example where the same mechanism is at work is the perfect boson gas. There is no phase transition in a gas with a non conserved number *N* of bosons, as in the case of photons, but if the number *N* of particles is fixed Bose–Einstein condensation happens. The partition function of the conserved bosons, for a given volume and temperature, has a singularity at a certain value of the boson number $N=N_{c}$ (or density). This singularity corresponds to the critical number of particles below which the condensed phase develops. According to the duality principle discussed above, this implies that the probability distribution of the number of bosons in a system of, say, photons, where this number is allowed to fluctuate, will be singular at the same value $N_{c}$ of the random variable *N* [33]. The very urn model is another instructive example. One can consider a model, dual to the one discussed in Section 3.3, where the total number of balls is conserved [21]. Marbles can only be exchanged among boxes and their density ρ is an external control parameter. This model is known to be interested, for k>2, by a phase transition crossing $\rho =\rho_{c}$. Notice that, since ρ is a control parameter, having $\rho >\rho_{c}$ in this dual model is not a rare event (as in the model introduced in Section 3.3). A similar situation is found in related models such as the zero range process [18,21,28].

### 4.2. Condensation

In order to see how singularities may come about in another perspective we will discuss the phenomenon in the framework of the urn models, where the physical meaning is probably more transparent in term of a condensation mechanism. Something similar occurs also in the other models considered in Section 3, regardless of the fact that the rate function is well defined or not.

**Figure 5:**
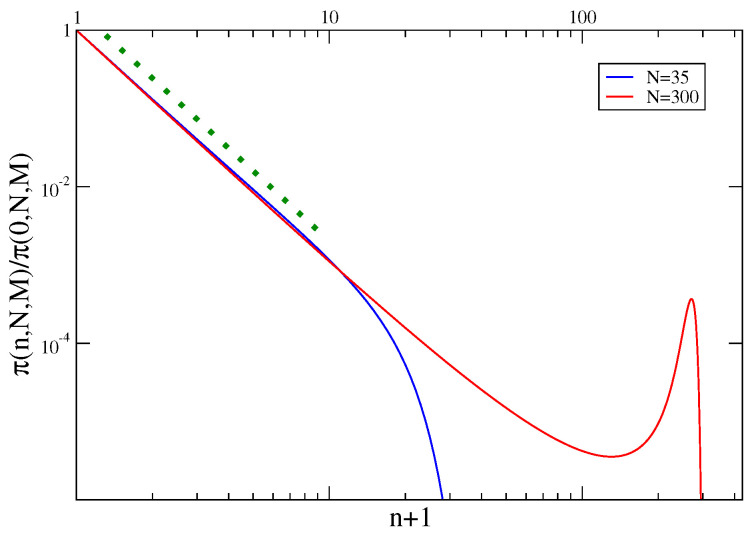
The function π(n,N,M) is plotted, for k=3 and M=100, against n+1 for two values of *N*: N=35, corresponding to a case without condensations, and N=300, corresponding to a condensed situation. The dotted green curve is the power-law $x^{-k}$.

Let us consider the conditional probability π(n,N,M) that one of the *M* a priori equivalent urns contains *n* particles, given that there are *N* particles in the whole system. This quantity can be evaluated exactly and is shown in Figure 5 (normalized by its value in n=0 to better compare curves with different *N* in a single figure). Let us discuss its properties. First of all π vanishes for n>N, since it is impossible that an urn contains more particles than the whole system. Secondly, for small *n* one has π(n,N,M)∝p(n) (dotted green line in Figure 5). This means that, as far as very few particles are stored in the tagged urn, the condition on the total number of balls is irrelevant.

More interestingly, at large *n*, n≲N, different behaviors are observed in the region of relatively small *N*, and in the one with relatively large *N*, exemplified by N=35 and N=300, respectively, in Figure 5. In the former case π is exponentially damped at large *n*, meaning that accommodating many particles in a single urn is probabilistically very unfavorable. In the latter case there is a peak at a value of *n* or of order *N*. This means that a significant fraction of the total number of particles is located in a single urn. This is the condensation phenomenon. (We will see in the next section that in this particular model occurs when k>2 and for sufficiently large densities). The essence of a condensation phenomenon is that a given quantity is not fairly distributed among many degrees of freedom, but is concentrated in a single one. This is particularly clear in the urn model, where one particular urn contains a macroscopic fraction of balls.

One easily realizes that something similar occurs in the other example models discussed in Section 3. For instance, in the model of Section 3.1, writing N[φ] in terms of the Fourier components $\varphi_{\vec{k}}$ of the field φ as

(17) $$N\left[\varphi \right]=\frac{1}{V}\sum_{\vec{k}}{\left|\varphi_{\vec{k}}\right|}^{2},$$

one can show that while for $N\leq N_{c}$ (or equivalently $\rho \leq \rho_{c}$) all the Fourier components add up to realize the sum in Equation (17) in a comparable way, for $N>N_{c}$ the term with k=0 alone provides the most important contribution to the sum. A similar mechanism, with the dominance of the k=0 term, is also at work in the example of Section 3.2. In the Maxwell–Lorentz particle model (Section 3.4) one has that normal entropy fluctuations are formed by the addition of many contributions associated to many short flights of the probe particle. However above the critical threshold $\rho_{c}$ they are associated to a single event which is responsible for a macroscopic contribution to the entropy production. This event is a long flight of the probe particle with no collisions with the scatterers. For more details and a very accurate analytical description of these kinds of behaviors in a similar system re-framed in the context of active particles, see the recent work [48].

### 4.3. Mathematical Mechanism

In the previous section we have discussed the phenomenon of condensation on physical grounds. In this section we show the underlying mathematical mechanism. We will give a description as simple as possible, without presumption of mathematical rigor, in the framework of the urn model.

The probability distribution of the total number of particles *N* reads

(18) $$P\left(N,M\right)=\sum_{n_{1},n_{2},\cdots ,n_{M}}p\left(n_{1}\right)p\left(n_{2}\right)\cdots p\left(n_{M}\right)\;\delta_{\sum_{m=1}^{M}n_{m},N}\;,$$

where $\delta_{a,b}$ is the Kronecker function and in the leftmost sum the variables $n_{1},n_{2},\cdots ,n_{M}$ run from 0 to *∞*. Using the representation

(19) $$\delta_{a,b}=\frac{1}{2\pi i}\oint dz\;z^{-(b-a+1)}$$

of the δ function one arrives at

(20) $$P\left(N,M\right)=\frac{1}{2\pi i}\oint dz\;e^{M[\ln Q(z)-\rho \ln z]},$$

where

(21) $$Q\left(z\right)=\sum_{n=0}^{\infty }p\left(n\right)z^{n},$$

and we have confused $\frac{N+1}{M}$ with ρ=N/M for large *M*. Still for large *M*, the integral in Equation (20) can be evaluated by the steepest descent method as

(22) $$P\left(N,M\right)≃e^{-MR(\rho )},$$

where

(23) $$R\left(\rho \right)=-\ln Q\left[z^{*}\left(\rho \right)\right]+\rho \ln z^{*}\left(\rho \right),$$

with $z^{*}$ the value of *z* for which the argument in the exponential of Equation (20) has its maximum value. This in turn is given by the following implicit saddle-point equation

(24) $$\Theta (z^{*})=\rho ,$$

where

(25) $$\Theta \left(z^{*}\right)=z^{*}\frac{Q^{\prime }\left(z^{*}\right)}{Q(z^{*})}.$$

**Figure 6:**
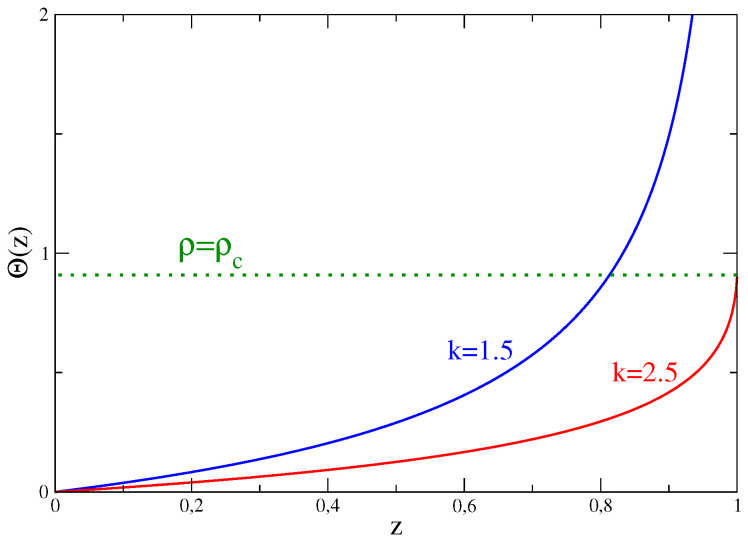
The function Θ(z) is shown for k=1.5 and k=2.5.

Let us study this equation. Clearly, it must be z≤1 in order for the sums hidden in *Q* and $Q^{\prime }$ to converge. It can also be easily seen that Θ(0)=0 and that this function increases with *z* up to

(26) $$\Theta \left(1\right)=\left\{\begin{array}{cc} \infty , & k\leq 2\\ \Theta_{M}, & k>2, \end{array}\right.$$

where $\Theta_{M}$ is a finite positive number. The function Θ(z) is shown in Figure 6, for two values of the parameter *k*. As it is clear from this figure, for k>2 the saddle point Equation (24) admits a solution only for $0\leq \rho \leq \rho_{c}=\Theta \left(1\right)$. It is trivial to show that $\rho_{c}\equiv \langle \rho \rangle =\sum_{n}np\left(n\right)$. However nothing prevents fluctuations with ρ>〈ρ〉 to occur. How can we recover the model solution for ρ>〈ρ〉? We know that for such high densities urns are no longer equivalent: there is one—say the first—which hosts an extensive number of particles and condensation occurs. In a physically oriented approach, we can take into account this fact by writing, in place of Equation (18), the following

(27) $$P\left(N,M\right)=M\sum_{n_{1}=0}^{\infty }p\left(n_{1}\right)\sum_{n_{2},n_{3},\cdots ,n_{M}}p\left(n_{2}\right)p\left(n_{3}\right)\cdots p\left(n_{M}\right)\;\delta_{\sum_{m=2}^{M}n_{m},N-n_{1}}.$$

The factor *M* in front of the r.h.s. stems from the fact that there are *M* ways to chose the urn (denoted as 1) to be singled out. Repeating the mathematical manipulations as in Equations (18) and (20), but only on the sum $\sum_{n_{2},n_{3},\cdots ,n_{M}}\cdots$, one arrives at

(28) $$P\left(N,M\right)=\frac{M}{2\pi i}\sum_{n_{1}=0}^{\infty }p\left(n_{1}\right)\oint dz\;e^{M[\ln Q\left(z\right)-\left(\rho -\frac{n_{1}}{M}\right)\ln z]}.$$

Evaluating the integral with the steepest descent method, the saddle point equation is now

(29) $$\Theta \left(z^{*}\right)=\rho -\frac{n_{1}}{M}.$$

Notice that in a normal situation, where condensation does not occur, in the thermodynamic limit where M→∞ with fixed ρ, the typical number of particles in a single urn does not depend on the number of urns. Therefore the last term on the r.h.s. of Equation (29) is negligible and one goes back to the previous saddle point Equation (24). However, when condensation occurs (i.e., with k>2 and ρ>〈ρ〉) the only possibility to close the model equations is to have the last term in Equation (29) finite. In conclusion one has

(30) $$\left\{\begin{array}{ccc} z^{*}<1, & \frac{n_{1}}{M}≃0 & \mathrm{no}\;\mathrm{condensation}\\ z^{*}=1, & \frac{n_{1}}{M}=\rho -\langle \rho \rangle & \mathrm{condensation}. \end{array}\right.$$

Clearly we are in the presence of a phase-transition resembling the ferro-paramagnetic or the gas–liquid transitions. There are two phases with qualitatively different behaviors. However, at variance with usual phase transitions, here the parameter producing the transition is not an external one that can be varied at will, but the value of the spontaneously fluctuating variable *N*. Another difference with usual phase transitions is the fact that here there is no interaction among urns. Despite that, urns are not completely independent due to the constraint over the number of particles represented by the Kronecker function in Equations (18) and (27). This constraint can be regarded as an effective interaction determining the transition (it can be easily seen, in fact, that without such conservation there is no transition).

Notice that it is $n_{1}/M=0$ in the normal phase and $n_{1}/M\neq 0$ in the condensed phase, therefore this quantity represents the order parameter of the transition. Despite the fact that a priori the system (i.e., the Hamiltonian) is invariant under a permutation of the urns, namely all boxes are equal, this property is not shared by the physical realization of the actual state of the system when condensation occurs, since one urn behaves very differently from the others. We are in the presence of spontaneous symmetry breaking.

As a final remark, let us note that the phenomenon of condensation in the sum of many identically distributed variables is not specific to an algebraic decay of p(n), or to the discrete value of the variable *n*. Indeed it is found [31] that it occurs provided that $\sum_{n}np\left(n\right)<\infty$. Condensation in the presence of a stretched exponential p(n), for instance, has been discussed in [59,60]. Finally, we mention the fact that in the context of Lévy flights the phenomenon of condensation is usually referred to as the big jump principle [61].

### 4.4. Fluctuation Relation

The Fluctuation Relation is one of the few general results of non-equilibrium statistical mechanics, expressing an asymmetry property of the fluctuations of some extensive (in time or in number of degrees of freedom) quantities *N* [40]. The FR reads

(31) $$\frac{P(N/M=\rho )}{P(N/M=-\rho )}=e^{cM\rho +o(M)},$$

where *c* is a constant, and o(M) stands for sub-linear corrections in *M*. Usually, the exponential form of the FR is related to two properties of P(N/M): (i) it satisfies a LDP Equation (5), and (ii) the rate function I(ρ) has the symmetry:

(32) I(−ρ)−I(ρ)=cρ.

These two conditions, with I(ρ) different from 0 and *∞*, are known to be sufficient for N/M to satisfy a FR (see, e.g., [4] and references therein).

It is interesting to consider the validity of an FR in the case of probability distributions with singularities. First, let us note that, when the singularity appears in zero, as in the case of the “kink” mentioned in Section 3.5, then the validity of an FR is clearly not affected by the singularity. More in general, the FR can also be satisfied by a pdf for which a standard (namely, with a leading exponential scaling in *M*) LDP does not hold. This can be observed for instance in the driven Maxwell–Lorentz gas described in Section 3.4. In this model it has been shown [11] that the entropy production calculated over a time *t* satisfies an FR, even though the far positive tail of its pdf scales exponentially with $\sqrt{t}$ rather than *t*. In this case the validity of the FR can be exploited to extract some information on the behavior of the probability distribution in the regions where the stretched-exponential scaling takes place.

The FR Equation (32) in the presence of a singular rate function has been also observed [37], besides the already mentioned Maxwell-Lorentz case , in some large time limit for the exchanged heat, in the large-N model of Section 3.2. More recently, it has been shown [39] that the rate function of the heat exchanged by a set of uncoupled Brownian oscillators with the thermostat during a non-stationary relaxation process does not satisfy an FR in the form Equation (31). Although, even in this case, the rate function shows a singular behavior in the limit of a large number of degrees of freedom, the lack of a standard FR is not necessarily related to the presence of the singularity.

## 5. Some Peculiarities of Singular Distributions

### 5.1. Giant Response

Generally, the behavior of a collective quantity such as the empirical mean Equation (1) is not substantially altered if, for large *M*, the properties of only one out of *M* variables is slightly modified. For instance, one does not expect to observe any significant change in the thermodynamic properties of a gas of identical molecules if one is replaced with another of a different substance. This is because the collective properties are determined by the synergic contribution of a huge number *M* of constituents, and hence the features of a single molecule are negligible. This is true not only for the typical properties but also for the fluctuation distribution. However, the situation can be dramatically different when singular probability distributions enter the game.

Let us show this with the prototypical example of the urn model. We consider a slightly modified version of the model defined in Section 3.3, where a single variable, say $n_{\ell }$, is distributed as in Equation (11) but with an exponent $k_{\ell }$ that may be different from the one, *k*, of all the remaining ones. Let us now look at Equations (28)–(30). In a situation where condensation does not occur, as we remarked earlier, the effect of a single variable is negligible, the first line of Equation (30) applies and hence $\frac{n_{m}}{M}≃0$, for any *m*. On the other hand, in the presence of condensation, the second line of Equation (30) holds. In the case of equally distributed variables condensation occurs with equal probability in any of the urns. However, if the *ℓ*-th variable behaves differently, one has to understand if the condensing variable could be the *ℓ*-th, or any of the remaining ones. Both the cases can occur, depending on the values of the exponents *k* and $k_{\ell }$.

For $k_{\ell }>k>2$ (the latter inequivalence being needed for condensation) it is $p\left(n_{\ell }=n\right)\ll p\left(n_{m}=n\right)$ for large *n* (with ℓ≠m). Hence the condensation phenomenon, which occurs by letting a huge amount of particles occupy a single urn, is unfavoured in the *ℓ*-th urn. The situation in this case is analogous to the one discussed before with equally distributed variables, i.e., with $k_{\ell }=k$. However for $k>k_{\ell }>2$ the opposite occurs, the condensing variable is the *ℓ*-th. Hence Equation (28) applies with $n_{1}$ replaced by $n_{\ell }$. One sees from Equation (28) that, when condensation occurs, P(N,M) is proportional to $p(n_{\ell })$. Since $k_{\ell }\neq k$, P(N,M) turns out to be different from the one found for equally distributed variables. Hence, in this case, an even small change of the properties of a single variable can trigger the form of the probability distribution of the collective variable *N*, a fact that is sometimes referred to as giant response.

**Figure 7:**
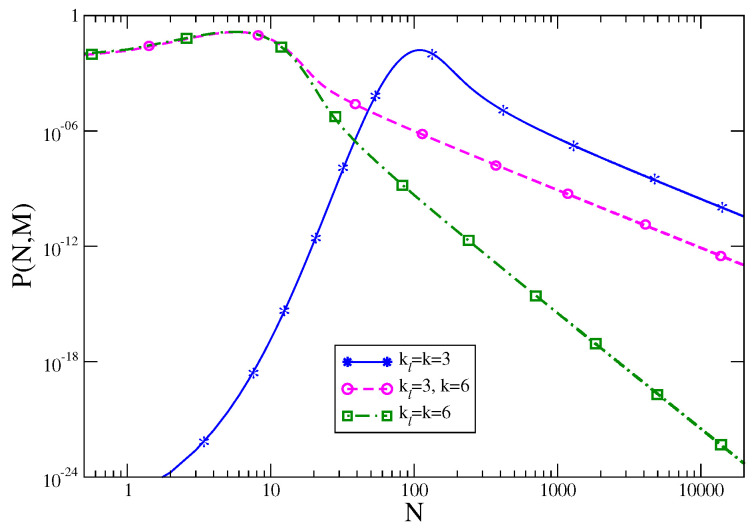
*P* is plotted for M=333 and the three different choices (see text) (i) $k_{\ell }\equiv k=3$, continuous blue with asterisks, (ii) $k_{\ell }\equiv k=6$, dot-dashed green with squares and (iii) $k_{\ell }=3$ , k=6, dashed magenta with a circles.

This is illustrated in Figure 7. Here P(N,M) is compared for three different choices of the exponents $k,k_{\ell }$. The continuous blue curve with asterisks refers to the case (i) with identically distributed variables with $k_{\ell }=k=3$. Similarly, the dot-dashed green curve with squares corresponds to the situation with (ii) $k_{\ell }=k=6$. Instead, the dashed-magenta curve with circles corresponds to non-identically distributed variables with (iii) $k_{\ell }=3$ and k=6. Notice that in the region to the left of the maximum, where condensation does not occur (because in this region ρ<〈ρ〉), the curves of the cases (ii) and (iii) coincide. This nicely shows that in the absence of condensation the shift of the properties of a single variable does not influence the collective behavior of the system. For ρ>〈ρ〉, on the other hand, the form of *P* drastically changes in going from (ii) to (iii), namely by perturbing the properties of one single variable. Even more impressive, the slope of the curve for case (iii) is the same as that of case (i), showing that this feature is dictated by the sole properties of the variable, $n_{\ell }$, which in case (iii) behaves as in (i).

### 5.2. Development of a Singular Fluctuation

We have seen in Section 4.1 that a singularity in the probability distribution can be interpreted as a phase transition occurring at a critical value of ρ, playing the role of a control parameter. The analogy can be pushed a step further. When a system is prepared in a certain equilibrium state and then a control parameter is changed as to make it cross a phase transition, the ensuing dynamics can be slow and characterized by a dynamical scaling symmetry associated most of the times with an ever growing length scale [62,63,64,65]. Typical examples are magnets and binary systems quenched across the critical temperature, and glassy systems.

Building on the analogy above, one might expect something similar to happen if one prepares a system with a singular P(N) in a state such that the fluctuating collective variable *N* takes a definite value $N_{0}$ on one side (say the left) of the critical value $N_{c}$ where the singularity takes place. If the system is then left to evolve freely, all possible fluctuations will take place, including those on the other side (say the right) of the singularity. Due to the duality principle, this process should occur in a way akin to the kinetics of a system brought across a phase-transition. Hence slow evolution and dynamical scaling should be observed. This has actually been shown to be the case, as we discuss below.

**Figure 8:**
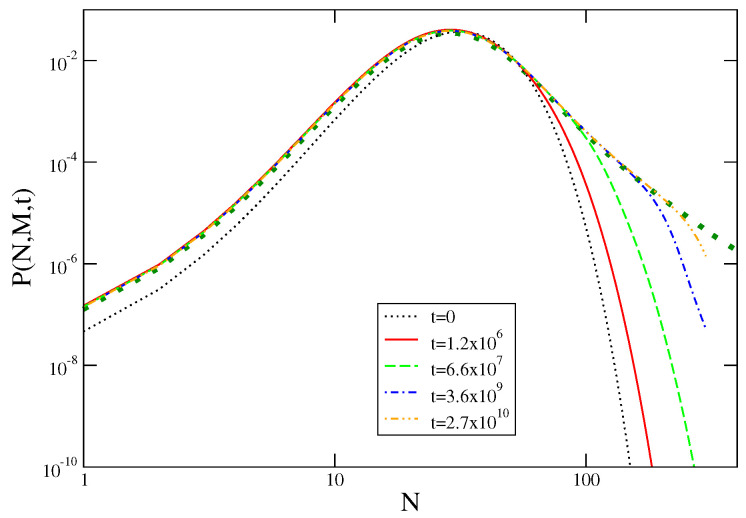
The probability P(N,M,t) with k=3 is plotted against *N* with double logarithmic scales for different times (see key), exponentially spaced. The dotted green line is the asymptotic form.

Upon supplementing the urn model of Section 3.3 with a kinetic rule allowing the system to exchange single particles with an external reservoir in such a way that the stationary occupation probability of any urn is given by Equation (11), one can solve exactly [43] the evolution of a system whose initial state is such that condensation is not present. In the following we will discuss the case in which the initial value of the density is ρ=〈ρ〉. Starting from this configuration, corresponding to an initial form P(N,M,t=0) of the probability distribution of the collective variable, the system will evolve as to produce all the allowed fluctuations. Hence P(N,M,t) becomes time-dependent. Clearly, for long times it is expected to approach the stationary value P(N), with the singular behavior already discussed. This curve is plotted in Figure 8, with a dotted green line.

In this figure one sees that the time evolution of the probability P(N,M,t) towards this asymptotic form is much different on the two sides of the singularity. For N<〈N〉, in the normal region without condensation, the evolution is fast and the asymptotic form of the probability is attained at relatively short times. Indeed, already the red curve for $t=1.2\times 10^{6}$ is indistinguishable from the stationary form and increasing time does not change anything. Conversely, the evolution is slow in the condensing region for $N>N_{c}$. Here one sees that, at any time, the asymptotic form is only attained up to a value N=ν(t), beyond which P(N,M,T) drops much faster than what expected asymptotically. It can be shown that ν(t) grows indefinitely in an algebraic way, much in the same way as a characteristic growing length does in systems quenched across a phase transition. In addition, a dynamical scaling symmetry can be shown to be at work also in this case. The origin of this slow kinetics is clearly due to the difficulty to condense a huge amount of particles in a single urn by exchanging single particles with the reservoir.

### 5.3. Observability

In the previous sections we discussed some peculiar properties of singular distribution functions. A natural question is if such features can be observed in practical situations. Indeed, the non-analycities of the probability distributions are observed in the regime of large deviations, namely outside the range of small fluctuations which are generally described by the central limit theorem and are more likely to be observed.

To make more clear this point let us make reference to the Gaussian model and, specifically, to Figure 1. In this case, in order to detect singular deviations, $\rho =\rho_{c}$ must be exceeded. Namely, the system has to move quite far from the most likely observed value—the maximum of the distribution. If the LDP Equation (5) holds (it does so in this model) the possibility to observe such a large fluctuation is extremely small already for moderately large values of the number of constituents *M* (or volume *V*), due to the exponential damping in *M* expressed by Equation (5). But the situation is different if the LDP is violated. This occurs, for instance, in the urn model or in the Maxwell–Lorentz gas, in the fluctuation range where the rate function vanishes. In the former model one can easily check from Equations (28)–(30) that the LDP is obeyed in the non-condensing regime but it is violated when condensation occurs. In fact, it is trivial to see that with $z^{*}=1$ the saddle point evaluation of the integral in Equation (28) gives an exponential with an argument that is identically vanishing. As a consequence fluctuations away from the average are no longer damped exponentially in *M*, but only as $M^{1-k}$ (keeping ρ fixed). This is why the rate function of the model vanishes in the whole sector $\rho >\rho_{c}$ where condensation occurs (see Figure 3), despite the fact that P(N,M) decays for ρ>〈ρ〉, as it can be seen in Figure 7. Due to this much softer decay, there is a better chance to observe singular fluctuations in this model than in others, e.g., the Gaussian model, where the LDP holds. A similar situation, with LDP violations, is observed also in the Maxwell–Lorentz particle model (for $\rho =\Delta s_{tot}/t>\rho_{c}$) [11] and in Bose–Einstein condensates [33].

## 6. Summary and Conclusions

In this paper we have shortly reviewed the issue of probability distributions characterized by non-analyticities. Naively, this feature could be considered as a rare manifestation of curious mathematical pathologies occurring in scholarly model with uncertain relations to the physical world. In reality, singular probability distributions have been shown analytically to occur in very simple and fundamental models of statistical mechanics, such as the Gaussian one, and not only in weird non-equilibrium states but also in equilibrium. Furthermore, they have been detected in numerical simulations and, most importantly, also in real experiments. This widespread occurrence points towards an underlying general mechanism for the development of singularities in the fluctuation probability. This paper has been conceived in order to highlight and discuss, at a simple and physically oriented level, at least some of such general features.

In the first part of the paper, after recalling basic and general concepts of probability theory we have reviewed some models where singular fluctuation spectra have been observed. These range from the aforementioned Gaussian model to the spherical limit of a ferromagnet, from the so-called urn model to a description of the Maxwell–Lorentz gas. In all these cases the deviations of certain collective observables are described by non-analytical probability distributions, which, in the case when LDP holds, are characterized by the presence of exponential branches.

The non-analytical behavior has been interpreted as due to the same mechanism whereby singularities develop in the thermodynamic functions of systems experiencing phase transitions. Indeed we have discussed the fact that a singular fluctuation distribution function can be mapped onto a thermodynamic potential of a dual model with a critical point. The singularity appears similarly to what one observes in thermodynamic functions when a condensation transition is present. When such feature occurs at the level of fluctuations, at variance with the usual examples of condensation, one speaks of condensation of fluctuations.

Singularities of the probability distributions can have a scarce practical relevance if they occur in regions where fluctuations have a negligible chance to be observed. However, in some of the cases considered in this paper the non-analytical behavior is associated to the breakdown of the large deviation principle. As a result, large fluctuations of macrovariables have a better chance to be observed even in systems with a relatively large number of degrees of freedom. In this case the presence of singularities not only can be observed, but its effects can be appreciated. Perhaps, one of the most intriguing one is the so called giant susceptibility, whereby slightly tuning the properties of even one single component, say a molecule of a gas, can have catastrophic consequences on the behavior of the whole system.

Non-analyticity points in the probability distributions also influence the way in which rare fluctuations are developed out of typical state where they are absent. Indeed, it has been shown that large fluctuations in the region where condensation occurs are formed by means of a complex slow dynamics which resembles, once again a manifestation of a dual behavior, that of systems brought across a phase transition. The knowledge of the dynamical path leading to a rare fluctuation may have important consequences in those cases when such deviations lead to catastrophic events, as in the case of extinctions or bankruptcies.

Among the several perspectives of future studies in this context, we mention the possibility to explore the role of correlated noise on the large deviations, for instance in models of active particles where some analytical results can be obtained [66]; the meaning of singularities, which are related to non-equilibrium phase transitions, within the general framework of the macroscopic fluctuation theory [67]; the relation between the presence of singularities and the validity of the fluctuation relation for entropy production or related quantities in more general cases; the role of correlations among random variables in the anomalous large deviations, as observed for instance in conditioned random walks [68] and Brownian motion [69]; the effect of inhomogeneous rates in bulk-driven exclusion processes [70].
